# M6A methylation modification in autoimmune diseases, a promising treatment strategy based on epigenetics

**DOI:** 10.1186/s13075-023-03149-w

**Published:** 2023-10-02

**Authors:** Yurong Huang, Qiuyun Xue, Jun Chang, Yuting Wang, Chenglong Cheng, Suowen Xu, Xiao Wang, Chenggui Miao

**Affiliations:** 1 Department of Pharmacology, School of Integrated Chinese and Western Medicine, Anhui University of Chinese Medicine, No. 1 Qianjiang Road, Xinzhan District, Hefei, 230012 Anhui Province China; 2 Department of Orthopaedics, the First Affiliated Hospital, Anhui Medical University, Hefei, 230032 China; 3 Anhui Public Health Clinical Center, Hefei, China; 4 Department of Endocrinology, Institute of Endocrine and Metabolic Diseases, The First Affiliated Hospital of USTC, Division of Life Sciences and Medicine, Clinical Research Hospital of Chinese Academy of Sciences (Hefei), University of Science and Technology of China, Hefei, 230027 China; 5 Department of Clinical Nursing, School of Nursing, Anhui University of Chinese Medicine, Hefei, China

**Keywords:** m6a methylation modification, Methylation regulation, Autoimmune diseases, Rheumatoid arthritis, Systemic lupus erythematosus

## Abstract

**Background:**

N6-methyladenosine (m6A) methylation modification is involved in the regulation of various biological processes, including inflammation, antitumor, and antiviral immunity. However, the role of m6A modification in the pathogenesis of autoimmune diseases has been rarely reported.

**Methods:**

Based on a description of m6A modification and the corresponding research methods, this review systematically summarizes current insights into the mechanism of m6A methylation modification in autoimmune diseases, especially its contribution to rheumatoid arthritis (RA) and systemic lupus erythematosus (SLE).

**Results:**

By regulating different biological processes, m6A methylation is involved in the pathogenesis of autoimmune diseases and provides a promising biomarker for the diagnosis and treatment of such diseases. Notably, m6A methylation modification is involved in regulating a variety of immune cells and mitochondrial energy metabolism. In addition, m6A methylation modification plays a role in the pathological processes of RA, and m6A methylation-related genes can be used as potential targets in RA therapy.

**Conclusions:**

M6A methylation modification plays an important role in autoimmune pathological processes such as RA and SLE and represents a promising new target for clinical diagnosis and treatment, providing new ideas for the treatment of autoimmune diseases by targeting m6A modification-related pathways.

## Introduction

The discovery of RNA modification has opened up a new field of gene expression regulation in eukaryotes known as "RNA epigenetics". N6-methyladenosine (m6A), the most abundant modified form of messenger RNA (mRNA), is at the forefront of biological and medical research. In addition to being distributed in mRNA, m6A is also found in microRNA (miRNA) and long noncoding RNA (lncRNA) in many non-coding RNAs (ncRNAs) [1, 2]. M6A was first discovered in 1974 and is the best characterized mRNA modification to date. M6A is the main form of methylation in mammalian mRNA [3, 4].

M6A modification is involved in various aspects of RNA metabolism, including transcription, pre-mRNA splicing and processing, pre-microRNA processing, nuclear export, translation, RNA stability, and decay [5–8]. The m6A modification of RNA is a dynamic and reversible process similar to DNA and histone methylation. This reversible RNA modification determines the fate of the modified RNA molecules at the post-transcriptional level, having an impact on practically all significant biological processes. However, disturbances to m6A modification can lead to serious pathological changes, including cancer, metabolic disease, and abnormal immune system activation [9].

In addition, m6A modification is involved in regulating the biological functions for a variety of immune cells, such as peripheral blood monocytes (PBMCs), macrophages, dendritic cells, and T cells [10]. Various immune cells play key roles in the pathological processes of autoimmune diseases. The potential role of m6A modification in autoimmune diseases may be realized through the regulation of different immune cells, but its specific role and related mechanisms remain unclear [11].

Autoimmune diseases have a variety of etiologies, but their exact pathogenesis is unknown. Potential precipitating factors include the emergence of autoantigens, abnormal immune regulation, cross-antigens, genetic factors, and environmental/lifestyle factors [12, 13]. Systemic autoimmune illnesses and organ-specific autoimmune diseases are two categories of clinical presentation for autoimmune diseases. Systemic autoimmune diseases are induced by extensive deposition of the antigen–antibody complex in blood vessel walls and other causes of organ damage, including SLE and Scleroderma [14]. Organ-specific autoimmune diseases mainly include RA, hyperthyroidism (AITD), Type 1 diabetes (T1DM), ulcerative colitis, multiple sclerosis (MS), and multiple cerebrospinal sclerosis [15].

RA is characterized by erosive arthritis, and its pathological basis is synovitis. In the early stages of the disease, the patient experiences joint swelling and pain, eventually leading to joint deformity; in addition, a loss of normal joint function can occur [16]. Angiogenesis, interstitial inflammatory cell infiltration, pannus development, synovial lining cell proliferation, and the destruction of cartilage and bone tissue are the main pathological features of RA [17]. SLE is characterized by multi-organ and multi-system damage, with a variety of antibodies represented by antinuclear antibodies in serum, which alternately indicate remission and acute attack. The etiology of this disease remains unknown. Studies have found that the disease may interact with individual genetic factors, sex hormone levels, environmental factors, and immune dysfunction [18, 19]. Other autoimmune diseases include MS, scleroderma, AITD, T1DM, primary Sjogren's Syndrome (pSS), celiac disease, and uveitis. MS is a chronic inflammatory demyelinating disease of the central nervous system [20], while scleroderma is a connective tissue disease characterized by localized or diffuse fibrosis, sclerosis, and atrophy of the connective tissue of the skin and internal organs [21]. AITD is closely related to autoimmune diseases such as Graves' Disease [22]. T1DM is a metabolic disorder syndrome mainly caused by the immune system's destruction of pancreatic β cells and characterized by hyperglycemia brought on by a complete lack of insulin [23]. PSS is a chronic systemic autoimmune disease characterized by progressive exocrine gland damage and lymphocyte proliferation [24]. Celiac disease is a primary malabsorption syndrome characterized by intestinal mucosal lesions caused by gluten intolerance in patients [25]. Uveitis is a general term for inflammation of the iris, ciliary bodies, and choroid tissue, with a complex etiology [26].

M6A plays a critical role in the immune system and may influence the pathogenesis of autoimmune diseases. However, to date, there have been few reports of m6A in autoimmune diseases. Furthermore, the development of drugs based on m6A modification is very rare. Currently, no drugs based on m6A methylation modification have been developed to treat autoimmune diseases. In this paper, to provide new insights into the etiology of autoimmune illnesses and identify possible epigenetic therapy targets, we systematically summarize research data on m6A modification in autoimmune diseases. We also assess the feasibility of m6A modification as a potential epigenetic therapy target in RA and SLE. In addition, we discuss current challenges in this field of research and anticipate future research directions that could contribute to the pathogenesis and treatment of autoimmune diseases.

## Dynamic m6A methylation modification mechanism

M6A-mediated gene expression plays an important role in a variety of cell functions and disease processes. Post-transcriptional modification affects gene expression by participating in RNA processing and metabolism and ultimately affects cell growth and differentiation, as well as various physiological and pathological functions.

**Fig. 1:**
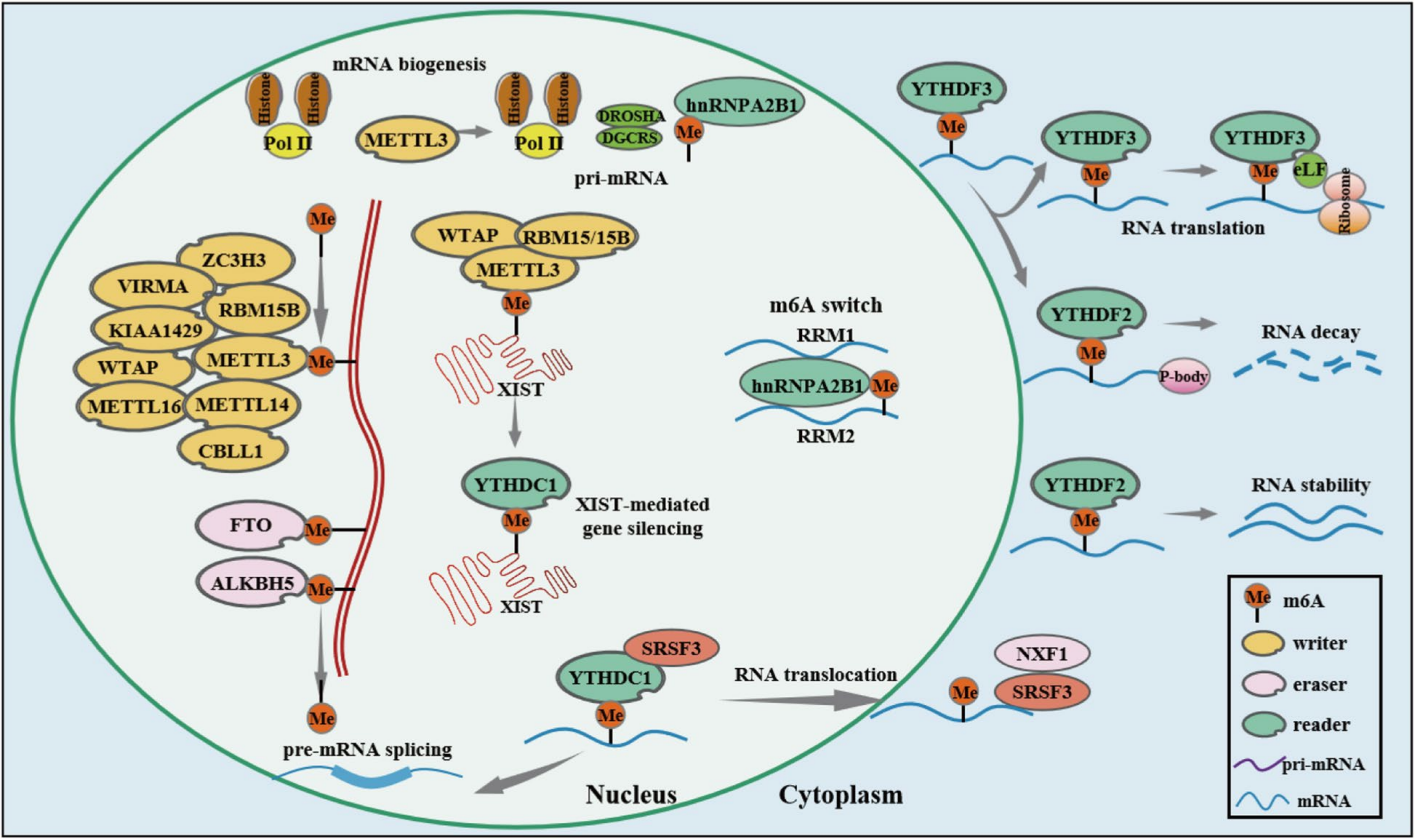
The roles of writers, erasers, and readers in the m6A modification of mRNA. The dynamic process of RNA adenine methylation is driven by three major groups of enzymes called writers, erasers, and readers, which are responsible, respectively, for methylation, demethylation, and decoding of the methylation code. The m6A methylation process is catalyzed by a writer complex, which includes METTL3, METTL14, METTL16, WTAP, VIRMA, RBM15/15B, CBLL1, KIAA1429, and ZC3H13. The m6A eraser demethylases include FTO and ALKBH5. The m6A readers are proteins that recognize and bind to m6A marks, affecting the fate and function of the modified RNAs. The known m6A readers are YTHDF1/2/3, YTHDC1/2, IGF2BP1/2/3, and HNRNPC/A2B1

**Fig. 2:**
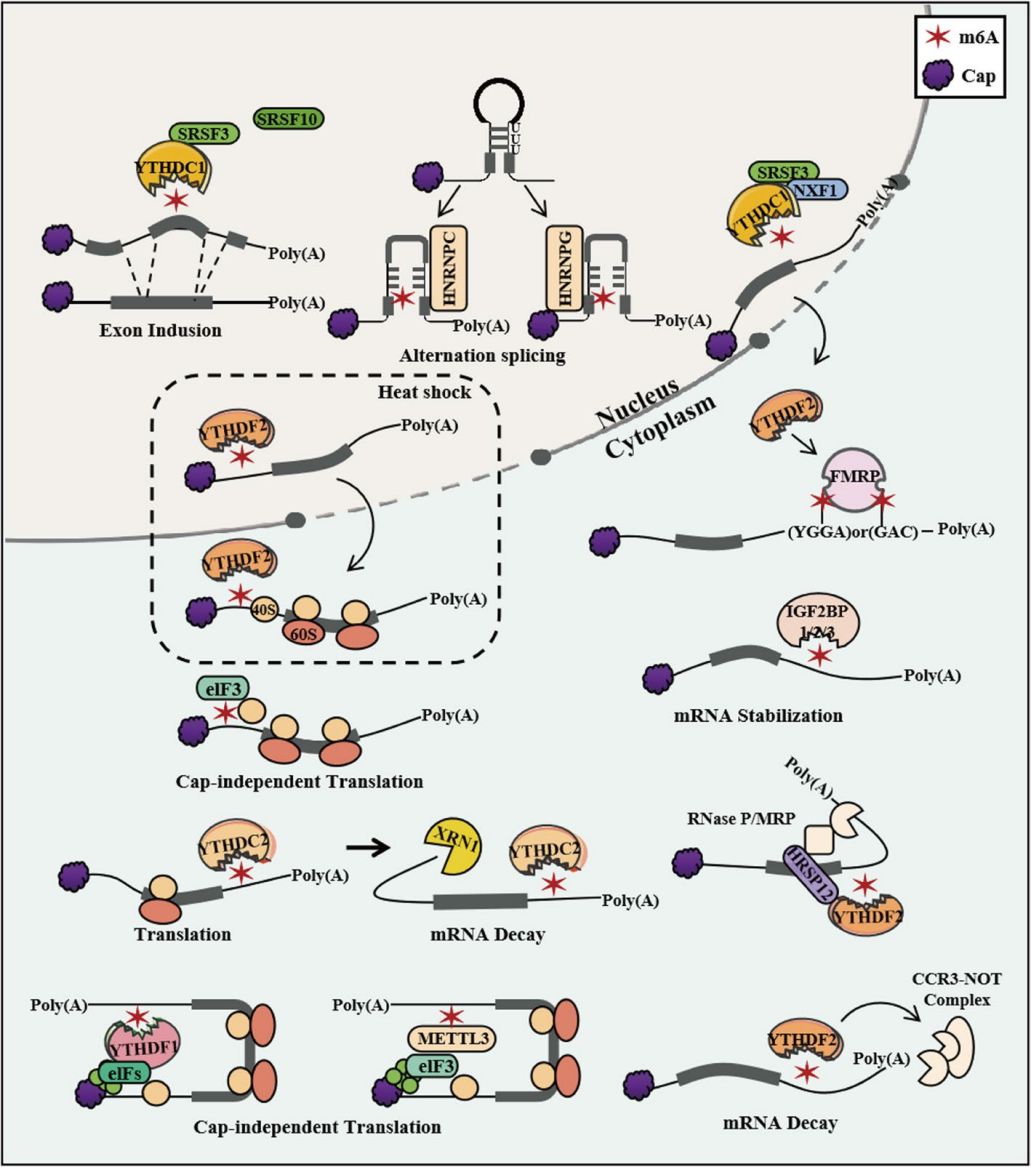
Molecular function of m6A in mRNA metabolism. Specific m6A-binding reading proteins regulate mRNA metabolism containing m6A, including m6A co-transcription modifications, m6A promoting alternative splicing, m6A promoting mRNA export, m6A altering RNA structure, m6A regulating translation efficiency, and m6A regulating mRNA stability. (The thick line represents the encoding sequence, and the thin line represents the UTR. The dashed box indicates the thermal shock state.)

The discovery of methyltransferase (also known as the m6A "writer") and demethylase (also known as the m6A "eraser") indicates that m6A modification is a dynamic and reversible event. M6A modification is controlled by so-called "writers", "erasers", and "readers" [27] (Table 1). The process of m6A methylation is catalyzed by a writer complex including methyltransferase-like 3 (METTL3), METTL14, METTL16, Wilms' tumor 1-associating protein (WTAP), vir-like m(6)A methyltransferase-associated protein (VIRMA), RNA binding motif protein 15 (RBM15)/15B, casitas B-lineage proto-oncogene like 1 (CBLL1), vir-like m6A methyltransferase-associated protein (KIAA1429), and zinc finger CCCH-type containing 13 (ZC3H13). The m6A erasers are demethylases and include the fat mass and obesity-associated (FTO) gene and AlkB Homolog 5 (ALKBH5). The m6A readers are proteins that recognize and bind to m6A marks, affecting the fate and function of the modified RNAs. The known m6A "readers" include the YTH domain family, member 1/2/3 (YTHDF1/2/3), YTH domain containing 1/2 (YTHDC1/2), insulin-like growth factor 2 mRNA binding protein 1/2/3 (IGF2BP1/2/3), and Heterogeneous nuclear ribonucleoprotein A2B1 (HNRNPC/A2B1) (Fig. 1). The steady-state balance of the m6A level in cells is maintained through the dynamic interactions between these three readers. The writer catalyzes the transfer of methyl from S-adenosylmethionine (SAM) to the N-6 position of adenosine (A) [28]. The eraser can erase methylation modification on the relevant site of the RNA, thus achieving demethylation modification. The reader can directly or indirectly act on RNA processing. Multiple readers dynamically regulate mRNA metabolism containing m6A, including alternative splicing, mRNA export, structural switching, translation, and mRNA stability, depending on the specific m6A-binding reading protein [29] (Fig. 2). These modifications are involved in the regulation of multiple biological processes, including inflammation and antitumor immune responses and antiviral immunity [30].

**Table 1:** The regulators involved in m6A methylation

Category	Genes	Function
Writers	METTL3, METTL14, WTAP, KIAA1492	METTL3 and METTL14 form complexes that catalyze the m6A methylation of RNA with WTAP and other factors such as KIAA1429.
Erasers	FTO, ALKBH5	The m6A modified base is demethylated by FTO and ALKBH.
Readers	YTHDC1, YTHDC2, YTHDF1, YTHDF2, YTHDF3	Recognizing RNA methylation modification information, participating in downstream RNA translation, degradation, and other processes.

The biological function of m6A depends on the involvement of various regulatory proteins. Over the past decade, numerous studies have explored the significance of m6A modification in diseases, and targeting m6A modification may have potential in therapeutic development. Recent studies have shown that abnormal m6A modification is associated with various pathophysiological processes in autoimmune diseases [31]. RNA methylation in autoimmune diseases such as RA and SLE involves the deposition of methyl groups, which is similar to the role of RNA methylation in cancer.

## Detection techniques of m6A methylation modification

With the upgrade of analytical chemical tools and the rise of high-throughput sequencing technology, it is becoming increasingly convenient to study RNA modification, including m6A-seq, MeRIP-seq, and single-base resolution detection (Table 2). In recent years, to quantitatively analyze m6A modification, researchers have abandoned the original direction of using m6A antibodies and developed methods for the identification of m6A without using antibodies: MAZTER-seq and DART-seq [32, 33]. MAZTER-seq mainly uses MazF RNA enzymes to cut RNA at the unmethylated ACA but cannot cut RNA at the methylated ACA. MAZTER-seq not only detects m6A sites de novo and quantifies methylation but also quantifies m6A in subcells, different cell types, and different disease states. In DART-seq, the cytidine deaminase APOBEC1 fuses with the YTH domain of m6A binding. DART-seq recognizes RNAs containing m6A throughout the transcriptome.

**Table 2:** M6A methylation detection methods

Methods	Characteristics	Resolution ratio	Technical shortcoming
MeRIP-seq/m6A-seq	Easy to do by using m6A antibody	200 nt	Relatively quantitative, low resolution, nonspecific binding
PA-m6A-seq	4-sU and RNase T1, high resolution	30 nt	Relatively quantitative, incomplete detection
M6A-CLIP/miCLIP	254 nm UV crosslinking	single base	Relatively quantitative, high RNA consumption
NT-m6A-seq	Nanopore technology, low capture error rate, and high accuracy of base recognition	single base	Relatively quantitative, high RNA consumption
MAZTER-seq	MazF RNA enzyme, quantitative analysis	single base	Limited applicable objects
DART-seq	Cytidine deaminase APOBEC1, quantitative analysis	single base	Complex operation

## M6A methylation in RA

**Fig. 3:**
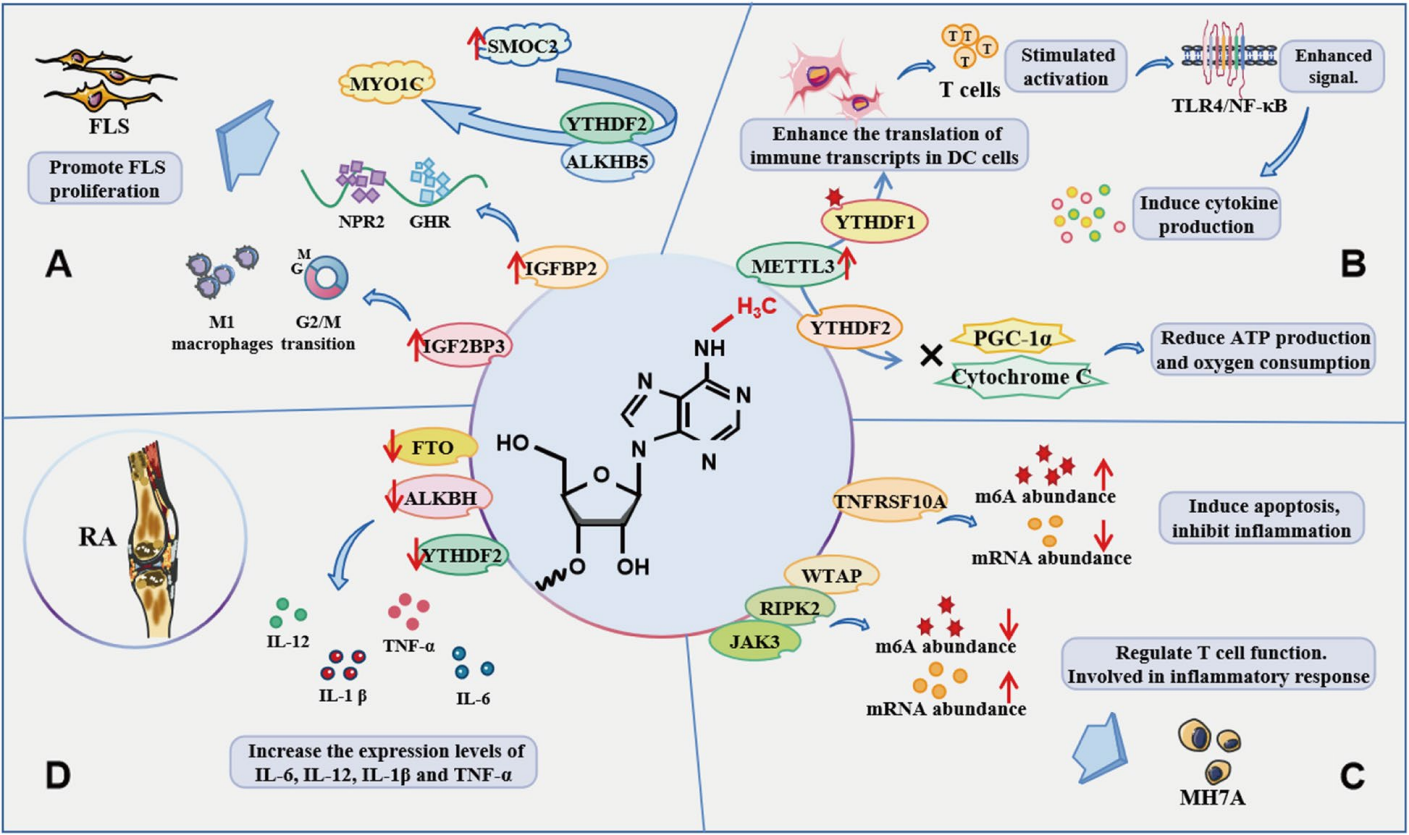
Genes with abnormal m6A methylation are involved in the pathogenesis of RA. **A** IGFBP2 influences the interaction between FLS by regulating the neuropeptides GHR and NPR2. IGF2BP3 regulates G2/M transition and affects the polarization of M1 macrophages. SMOC2 controls MYO1C expression through ALKBH5-mediated m6A modification. In RA FLS, these genes promote FLS proliferation through different pathways. **B** METTL3 promotes FLS activation and inflammation through the NF-κB signaling pathway. METTL3 and YTHDF2 synergically inhibited the expression of PGC-1α and cytochrome C and reduced the ATP production and oxygen consumption rates. **C** In MH7A cells activated by TNF, the mRNA abundance and m6A methylation abundance of WTAP, RIPK2, JAK3, and TNFRSF10A significantly changed. These genes are involved in the pathogenesis of RA through m6A methylation. **D** The low expression of ALKBH5, FTO, and YTHDF2 in RA peripheral blood is associated with changes in inflammatory markers and some key pro-inflammatory cytokines. These genes may be biomarkers or predictors for the assessment of RA onset, disease progression, and disease severity

The initial lesions of RA include microvascular system damage and the proliferation of fibroblast-like synoviocytes (FLS) arranged on the synovial membrane. FLS secrete metalloproteinases, chemokines, and inflammatory cytokines [34]. Macrophages, natural killer cells, dendritic cells, lymphocytes, and mast cells are RA-related immune cells that interact with inflammatory cytokines [35]. M6A methylation modification is involved in the regulation of a variety of immune cells. Abnormal genes associated with m6A methylation may be biomarkers or predictors for assessing the onset, progression, and severity of RA and are also expected to be potential targets for RA treatment (Fig. 3). Clarifying the role of m6A modification in RA will help us further discover a new pathogenesis of RA and provide a theoretical basis for the targeted therapy of RA.

### The role of m6A regulators in RA pathology

FLS plays an important role in the development of RA [36]. Insulin-like growth factor binding protein 2 (IGFBP2) plays an integral role in modifying the action of insulin-like growth factors in many cell types. IGFBP2 is elevated in RA FLS and influences the interactions between FLS by regulating the neuropeptide growth hormone receptor (GHR) and Recombinant Natriuretic Peptide Receptor 2 (NPR2), thereby contributing to the disease progression of RA [37]. Insulin growth factor-2 binding protein 3 (IGF2BP3) is a highly conserved paralog of IGF2BPs and also an m6A reader. In RA FLS, siIGF2BP3 decreased the expression of cyclin B1 (CCNB1) and cellular myelocytomatosis viral oncogene (c-Myc). IGF2BP3 can regulate the G2/M transition, promote the proliferation of RA FLS, and affect the polarization of M1 macrophages [38]. However, it remains to be confirmed how IGF2BP3 regulates macrophage polarization and inflammatory deterioration during RA progression through CCNB1 and c-Myc.

Modular calcium-binding protein 2 (SMOC2) increased significantly in RA FLS and synovial tissue [39]. Cytoskeleton-related genes are significantly down-regulated in RA FLS with low expression of SMOC2, especially the motor protein myosin 1c (MYO1C). SMOC2 controls MYO1C expression through ALKBH5-mediated m6A modification. Knockout of YTHDF2 could save the expression of MYO1C in RA FLS after the knockout of ALKBH5. When SMOC2 is silent in RA FLS, MYO1C can be modified via m6A mediated by ALKBH5 through YTHDF2-dependent pathway post-transcriptional regulation [40]. These studies indicate that ALKBH5 and YTHDF2 play key roles in RA, providing new insights into the pathogenesis of RA.

Targeting immune cells is one of the most effective ways to treat rheumatoid arthritis. M6A methyltransferase METTL3 alleviates RA by regulating cytokine changes and has the potential to treat immune diseases. The METTL3-mediated m6A methylation of certain immune transcripts enhances the translation of those transcripts in dendritic cells via YTHDF1, thus stimulating T cell activation and enhancing TLR4/NF-κB signaling-induced cytokine production [41]. METTL3 expression in PBMCs and FLS is significantly increased in RA patients compared with normal controls. M6A modification controlled by METTL3 is associated with cytokines IL-6 and TNF-α. Furthermore, METTL3 attenuates LPS-induced inflammatory responses in macrophages through the NF-κB signaling pathway [42]. METTL3 knockdown also inhibits IL-6, MMP3, and MMP9 levels in RA FLS and rat AIA-FLS. The marker protein (p)-p65 for the activation of the NF-κB signaling pathway is significantly down-regulated after METTL3 silencing and up-regulated after METTL3 overexpression [43]. METTL3 promotes FLS activation and inflammation through the NF-κB signaling pathway, which is an important mechanism in RA pathogenesis.

Mitochondrial biogenesis and energy metabolism disorder is one of the causes of RA, and peroxisome proliferator-activated receptor γ coactivator 1-α (PGC-1α) can control this process [44]. METTL3 and YTHDF2 synergically inhibit the expression of PGC-1α and cytochrome C and reduce ATP production and oxygen consumption rates, thereby increasing the accumulation of reactive oxygen species in cells and mitochondria and the level of pro-inflammatory cytokines in inflammatory monocytes. These results may provide new insights into the role of METTL3-dependent m6A-modified PGC-1α mRNA in monocyte inflammatory responses [45].

By high-throughput sequencing combined with methylated RNA immunoprecipitation (MeRIP-seq) and RNA sequencing, m6A-modified target genes were found to be closely related to RA inflammation and macrophages. The expression of m6A modified significantly transcriptor-rich inflammatory signaling pathway was evaluated through animal experiments, and it was found that the differential expression of m6A gene is closely related to macrophage activation and inflammatory processes such as MAPK, PI3K-Akt and JAK-STAT signaling pathways. Some genes enriched in the PI3K-Akt and MAPK pathways are mostly inhibitors of the corresponding pathways, including PTEN, ASPM and SHCBP1 [46, 47]. Through data mining, Liu et al. [48] demonstrated that the m6A-RNA methylation regulators (m6A-RMRs) were more differentially expressed in rheumatoid arthritis (RA) and autoimmune skin diseases than those in other autoimmune diseases. The identification of m6A methylation in RA will help find new intervention targets for the treatment and prevention of RA.

WTAP methyltransferase influences T cell functions, thereby contributing to the emergence of immunological disorders [49]. As a downstream target of the pattern recognition receptors NOD1 and NOD2, Receptor-interacting serine-threonine kinase 2 (RIPK2) is involved in inflammatory disorders [50]. Apoptosis is induced by TNFRSF10A, which also reduces inflammation, and the NF-κB pathway is activated by its ligand [51]. In contrast to the control group, WTAP, RIPK2, and JAK3 feature considerably less m6A methylation and more mRNA in MH7A cells activated by TNF. While TNFRSF10A's mRNA abundance is noticeably lower, its m6A methylation abundance is higher [52]. Through m6A methylation, these genes are involved in the pathogenesis of RA.

The expression of ALKBH5, FTO, and YTHDF2 changes significantly in the peripheral blood of RA, which is related to the production of autoantibodies and disease activity. The expression of YTHDF2 mRNA in the PBMCs of RA patients is low. Additionally, the expression of YTHDF2 mRNA is negatively correlated with ESR and CRP levels, the WBC count, and the NLR value [53]. Low expression of YTHDF2 increases the expression levels of IL-6, IL-12, IL-1β, and TNF-α induced by LPS. FTO and ALKBH5 are involved in mediating methylation reversal. Additionally, the overall m6A content in RA peripheral blood was found to be significantly higher than that in the healthy control group, and an increase in m6A content was negatively correlated with a decrease in FTO mRNA expression [54]. The low expression of ALKBH5, FTO, and YTHDF2 in RA peripheral blood is associated with changes in inflammatory markers and some key pro-inflammatory cytokines, which may be potential targets for regulating the inflammatory responses of RA.

CircRNA can affect the m6A methylation process. Circ_0066715 is expressed at low levels in RA, and the overexpression of miR-486-5p significantly inhibits the weakening of cell functions caused by the positive expression of circ_0066715 and the corresponding effects on M1 macrophage polarization. Circ_0066715 regulates the miR-486-5p/ETS1 axis, and the m6A methylation level of ETS1 is increased by WTAP knockout. The m6A methylation level of ETS1 increases after the knockout of WTAP, while the m6A methylation level of ETS1 decreases after the overexpression of WTAP [55]. These studies suggest a new circRNA/miRNA/mRNA regulatory axis and m6A regulatory mechanism involved in the polarization processes of RA macrophages, thus providing a powerful diagnostic and therapeutic strategy for RA treatment.

### The role of m6A methylation in RA treatment

As the m6A reader, IGF2BP3’s main mechanism of activity is based on its complex interactions with the miRNA mechanism. Both RNA binding proteins (RBPs) and miRNAs converge on the 3'-UTR of mRNAs. This binding contributes to the combinatorial mechanism of post-transcriptional gene regulation and has corresponding effects on cell fate and behavior [56, 57]. However, studies on IGF2BP3 in RA are very rare. Triptolide (TP) is the main active ingredient in Tripterygium wilfordii Hook F (TwHF), which was proven to have a therapeutic effect on RA [58]. Fan et al. [59] found that TP and IGF2BP3 have high binding affinity through molecular docking. In vitro experiments showed that TP could decrease the mRNA expression of IGF2BP3 in PBMCs and MH7A. Although IGF2BP3 may be a new target for TP in the treatment of RA, how TP affects RA through IGF2BP3 remains to be further studied.

Sarsasapogenin (Sar) extracted from the Chinese medicinal material Anemarrhena asphodeloides Bunge is a representative anti-inflammatory drug that can effectively improve the inflammatory responses of RA [60]. M6A methylation-mediated transglutaminase 2 (TGM2) promotes RA FLS proliferation by inducing DNA replication and cell cycle transition and inhibiting apoptosis through the activation of NF-κB signaling. Sar can disrupt the m6A methylation of TGM2, down-regulate TGM2 expression, inhibit FLS proliferation, and improve RA progression by disrupting the NF-κB signaling pathway [61]. TGM2 may be an attractive target able to provide new insights into the RA resistance mechanisms of Sar. Elucidating the mechanism of m6A modification in RA could help predict the effectiveness of immunotherapy strategies.

## M6A methylation in SLE

SLE is a complex autoimmune disease with multisystem involvement. In recent years, with the advancement of genetic, gene expression, and epigenetic research, the understanding of SLE has increased [62]. However, determining the exact mechanism of m6A modification in SLE requires further analysis.

One of the most important characteristics of SLE is the production of a variety of autoantibodies, such as anti-double-stranded DNA (anti-dsDNA). In addition, the ALKBH5 level is negatively correlated with anti-dsDNA level [63]. In SLE, low expression of METTL14 is associated with white blood cell and monocyte count, and low expression of YTHDF2 is associated with inflammation and fever [64]. The above genes can be used to evaluate SLE disease activity.

T cells are a core component of SLE pathogenesis. T cell homeostasis is a key process to maintain the size of the T cell pool, and its imbalance affects the onset of SLE [65]. Overexpression of ALKBH5 promoted apoptosis and inhibits T cell proliferation, thus affecting the disease activity of SLE. However, ALKBH5 is underexpressed in the PBMCs and T cells of SLE patients [66].

Through bioinformatics identification, IGFBP3 and two key immune genes (CD14 and IDO1) in SLE were found to be helpful in the diagnosis and treatment of SLE [67]. DNA hypomethylation may induce the onset of, or exacerbate, SLE. The expression of m5C and NSUN2 in SLE CD4 + T cells significantly decreased [68]. These studies provide a valuable new perspective for future exploration of the versatility and post-transcriptional significance of m6A modifications in SLE.

Examining the potential function of m6A modification targeting lncRNA in innovative therapeutic or diagnostic approaches for SLE will help uncover the potential role of m6A-related lncRNA in SLE. Two m6A-related lncRNAs (Xist and PSMB8-AS1) are down-regulated in patients with SLE and associated with several clinical manifestations of SLE [69]. Moreover, the level of circGARS in SLE is significantly up-regulated. MiR-19a-3p regulates the degradation of TNFAIP3 by down-regulating the expression of YTHDF2 through direct binding to circGARS. CircGARS modulates the expression of TNFAIP3 and influences the NF-κB pathway-mediated immunoinflammatory response in SLE [70]. It still remains to be determined how the relationship between m6A and RNA is regulated biologically. Our understanding of the pathogenesis of SLE will be improved by further research into the function of m6A alteration and epigenetics.

## M6A methylation in other autoimmune diseases

### M6A methylation in pSS

PSS is a multifactorial disease, and its potential genetic susceptibility, epigenetic mechanisms, and environmental factors all contribute to its occurrence [71]. Epigenetic mechanisms such as m6A affect the pathogenesis of pSS by regulating gene expression. METTL3, FTO, YTHDF1, and YTHDF2 mRNA are highly expressed in the peripheral blood of pSS patients and positively correlated with C-reactive protein (CRP) [72]. After targeting of these genes stimulates the expression of ISG15, the type I IFN signaling pathway is activated, which plays a positive role in initiating pSS autoimmunity.

Bioinformatics technology is helpful in understanding the potential regulatory mechanism of the m6A regulatory factor in pSS. Bioinformatic analysis was used to evaluate 23 m6A regulatory-mediated RNA modification patterns in the parotid glands and blood samples of pSS patients. The negative correlation between YTHDF2 and neutrophils in blood samples was the highest, and the correlation between FMR1 and mast cells showed the highest negative value. FMR1 was most significant in blood samples, and FMR1 and HNRNPC were most significant in parotid samples [73]. Cheng et al. [74] found that four m6A regulators in pSS (ALKBH5, RBMX, RBM15B, and YTHDF1) were down-regulated in peripheral blood, and four m6A regulators (ALKBH5, METTL3, RBM15B, and YTHDF1) were down-regulated in labial glandular tissue. ALKBH5 and METTL3 were fully connected with infiltrating immune cells and involved in the immune infiltration and autophagy of pSS, which may represent a new therapeutic target.

### M6A methylation in AITD

AITD is a thyroid disease caused by autoimmune disorders, autoantibodies against thyroid antigens can be detected in the blood of patients. The ALKBH5 gene in m6A modification acts on a variety of diseases [75]. Song et al. [76] detected five ALKBH5 gene variants in patients with AITD. Furthermore, high-throughput sequencing technology was used to detect the distribution of METTL3 genotypes in seven loci of AITD patients, and METTL3 gene polymorphism was associated with AITD susceptibility risk [77]. ALKBH5 and METTL3 are candidate genes for AITD susceptibility, making therapeutic targets potential.

### M6A methylation in Graves’ Disease

Graves' disease is a major clinical subtype of autoimmune thyroid diseases (AITDs) [78]. Through a high-throughput microarray, METTL3 and cytokine signaling (SOCS) molecules were abnormally expressed in the CD4 T cells of Graves' disease. After METTL3 knockdown, the expression of some factors of SOCS family members increased [79]. Inducing m6A methylation in members of the SOCS family is the mechanism by which METTL3 is involved in the development of Graves' disease.

### M6A methylation in T1DM

Immune-cell-mediated cell dysfunction is the root cause of T1DM [80]. In T1DM patients, METTL3 and IGF2BP2 are expressed at low levels, and YTHDC1, HNRNPA2B1, and HNRNPC are highly expressed. Gene Ontology (GO) and Kyoto Encyclopedia of Genes and Genomes (KEGG) pathway analyses demonstrated that differentially methylated transcripts were involved in pathways related to immunity, including some closely associated with T1DM [81]. Chen et al. [82] found that three single nucleotide polymorphisms (SNPs) in the non-coding regions PRRC2A (rs2260051 and rs3130623) and YTHDC2 (rs1862315) were significantly correlated with a risk of T1DM. PRRC2A was highly expressed in the peripheral blood of T1DM patients and involved in the interactions of cytokines with their receptors, cell adhesion and chemotaxis, and neurotransmitter regulation. The regulation of PRRC2A expression may be the mechanism underlying SNP-induced TIDM.

Cognitive impairment is a serious diabetes-related complication whose specific mechanisms remain unclear [83]. A T1DM mouse model established by intraperitoneal injection of streptozotocin (STZ) shows significant cognitive dysfunction [84]. The protein expression of YTHDF1, YTHDF3, and WTAP in the hippocampus of STZ group decreased. However, the cognitive impairment of STZ mice significantly improved after YTHDF1 overexpression [85]. YTHDF1 is a promising therapeutic target for diabetes-induced cognitive dysfunction.

### M6A methylation in MS

In all stages of MS, active demyelination and neurodegeneration are associated with inflammation mediated by T cells, B cells, macrophages and activated microglia. Multiple mechanisms contribute to neurodegeneration in progressive MS, including exhaustion of functional compensation, lack of trophic support, chronic microglial activation and altered expression of ion channels in demyelinated axons [86]. Approximately 80–85% of patients with MS experience a natural course of relapse and remission at disease onset that is referred to as relapsing–remitting MS (RRMS). The m6A modification in cerebrospinal fluid (CSF) can be used as a new potential diagnostic biomarker for PMS. The researchers identified differential expression of m6A regulatory genes between MS patients and non-MS patients, with m6A RNA methylation regulators upregulated in all 13 centers in MS patients compared to non-MS patients. Moreover, the m6A methylation level of RRMS samples was significantly higher than that of PMS samples in the analysis of cerebrospinal fluid samples [87].

By integrating data from large-scale GWAS and quantitative trait locus (QTL) studies using Mendelian randomization methods based on aggregated data, Mo et al. identified the mRNA expression of 178 DNA methylation sites and 29 MS-related genes. Among the identified non-MHC genes, METTL21B, METTL1 and TSFM had a strong association. Ms-related SNPS in DDR1 are closely related to plasma MHC Class I peptide associated Sequence B (MICB) and granzyme A levels. There is A causal relationship between plasma MICB and granzyme A levels and MS. Moreover, the interactions between DDR1, MICB, and GZMA and between METTL21B, METTL1, and TSFM act in the pathogenesis of MS [88].

Experimental autoimmune encephalomyelitis (EAE) is a widely cited animal model of multiple sclerosis [89]. ALKBH5, the m6A "eraser", may play a special role in controlling the pathogenicity of CD4 + T cells as an m6A eraser in the autoimmune process. The expression of ALKBH5 is up-regulated specifically upon T cell activation, and ALKBH5 controls CD4 T cell ability to induce autoimmune colitis. During the induced neuroinflammation, ALKBH5 deficiency increased m6A modification on interferon-γ (IFN-γ) and C-X-C motif chemokine ligand 2 (CXCL2) mRNA, thus decreasing their mRNA stability and protein expression in CD4 T cells. These modifications resulted in attenuated CD4 T cell responses and diminished recruitment of neutrophils into the central nervous system. Lack of ALKBH5 suppresses the IL-17 signaling pathway in CD4 T cells during EAE, T cell–specific deletion of ALKBH5 confers protection against EAE [90]. ALKBH5 may play a special role in controlling the pathogenicity of CD4 + T cells as an m6A eraser in the autoimmune process.

### M6A methylation in Celiac disease

Historically, the only effective treatment for people with celiac disease has been a strict lifelong gluten-free diet [91]. However, in a previous study, a novel m6A-XPO1-NF-κB pathway was found to be activated in patients with celiac disease. The XPO1 associated with celiac disease is preferentially methylated, and the translation of XPO1 depends on m6A and is mediated by the YTHDF1 protein. Gliadin can induce activation of the m6A mechanism in intestinal epithelial cells, resulting in increased XPO1 levels. Celiac patients exhibit allele-specific variations in XPO1 protein levels, increasing gluten-dependence in m6A, XPO1, METTL3, and YTHDF1, as well as downstream inflammatory effects [92]. These findings could support the development of new treatments targeting the m6A protein and XPO1, a potential therapeutic target for celiac disease.

### M6A methylation in uveitis

Uveitis is an inflammatory disease of the eye, and the role of m6A in this disease has been rarely reported. FTO expression was found to be decreased in the RPE cells of mice with experimental autoimmune uveitis. After FTO knockout, the m6A of ARPE-19 cells increased overall, while ATF4 protein expression decreased, and the m6A level of ATF4 increased. In addition, the cell proliferation and secretion of IL-6, IL-8, and MCP-1 increased after FTO knockout [93]. By regulating the m6A abundance of ATF4, FTO interfered with its mRNA translation and affected the release of inflammatory factors, leading to uveitis.

### M6A methylation in scleroderma

The cause of scleroderma is unknown. A mouse model of scleroderma was established by the subcutaneous injection of bleomycin (BLM) [94]. MeRIP-qPCR results showed that the methylation of Hras, Lama3, and Tnc in the BLM group was significantly increased, while methylation of Ccl3, Ccl9, Saa1, and Il1b significantly decreased [95]. M6A methylation is closely associated with fibrosis and inflammation regulation.

### M6A methylation in autoimmune hepatitis (AIH)

AIH is an immunoinflammatory chronic liver disease with dynamic and rather heterogeneous disease manifestations. Bone-marrow-derived suppressor cells (MDSC) are key elements of the innate immune system and an important driver of the subsequently acquired immune response to AIH [96]. YTHDF2 expression is significantly up-regulated in AIH patients. In YTHDF2 knockout mice, the absence of YTHDF2 resulted in a gradual elevation of MDSC in the liver. Notably, the lack of YTHDF2 in bone marrow cells can attenuate canavalin-induced liver injury and enhance liver expansion and chemotaxis. The inhibition of YTHDF2 can enhance the expansion, chemotaxis, and inhibitory functions of MDSC. Overall, YTHDF2 may represent a unique therapeutic target for immune-mediated hepatitis [97] Table 3.

**Table 3:** Regulators associated with M6A methylation in autoimmune diseases

Disease	The m6A regulators	Type	Sample source	Expression change	Regulatory roles	Reference
RA	IGFBP2	Reader	FLS	Up-regulation	Regulates the neuropeptides GHR and NPR2 to influence the interaction between FLS.	[37]
RA	IGF2BP3	Reader	Synovial tissues	Up-regulation	Regulates the G2/M transition and affects the polarization of M1 macrophages.	[38]
RA	SMOC2	-	Synovial tissues	Up-regulation	Controls MYO1C expression through ALKBH5-mediated m6A modification.	[40]
RA	METTL3	Writer	PBMC	Up-regulation	Attenuated LPS-induced inflammatory response in macrophages via the NF-κB signaling pathway.	[41]
RA	METTL3	Writer	FLS	Up-regulation	Activates the NF-κB signaling pathway.	[43]
RA	WTAP	Writer	MH7A cells	Up-regulation	Combines with METTL3 to form the MettL3–MettL14–Wtap complex.	[52]
RA	RIPK2	-	MH7A cells	Up-regulation	Mediates LPS-induced p38 and IKB-α signaling.	[52]
RA	JAK3	-	MH7A cells	Up-regulation	Mediates and participates in the formation of cytokines.	[52]
RA	TNFRSF10A	-	MH7A cells	Down-regulation	Activates the NF-κB pathway and promotes cell proliferation and migration.	[52]
RA	YTHDF2	Reader	PBMC	Down-regulation	Affects the production of autoantibodies.	[53]
RA	FTO	Eraser	Peripheral blood	Down-regulation	Mediates methylation reversal.	[53]
RA	ALKBH	Eraser	Peripheral blood	Down-regulation	Mediates methylation reversal.	[53]
SLE	ALKBH5	Eraser	Peripheral blood	Down-regulation	Affects anti-DSDNA levels	[63]
SLE	MTEEL14	Writer	Peripheral blood	Down-regulation	Perturbs the m6A threshold, leading to uncontrolled expression/activity of the virulence gene.	[64]
SLE	YTHDF2	Reader	Peripheral blood	Down-regulation	Perturbs the m6A threshold, leading to uncontrolled expression/activity of the virulence gene.	[64]
pSS	METTL3 FTO YTHDF1 YTHDF2	Writer Eraser Reader Reader	PBMC	Up-regulation	Targets ISG15, stimulates ISG15 expression, and activates the type I IFN signaling pathway.	[72]
Graves’Disease	METTL3	Writer	CD4T cells	Down-regulation	Induces SOCS mRNA m6A modification.	[79]
MS	ALKBH5	Eraser	T cells	Up-regulation	Controls CD4 T cell pathogenicity.	[89]
Uveitis	FTO	Eraser	EAU mouse RPE cells	Down-regulation	Regulates the m6A level of ATF4 to affect the translation of ATF4 and the expression of inflammatory factors.	[92]
Autoimmune hepatitis	YTHDF2	Reader	liver	Up-regulation	Low expression of YTHDF2 can enhance the amplification, chemotaxis, and inhibition of MDSC.	[96]

## Conclusions and perspectives

The regulation of m6A levels is complex and diverse. M6A methylation plays a broad and important role through the functional interaction between m6A methyltransferase, demethylase, and m6A binding protein [98]. The misexpression of any factor in this network can lead to abnormal methylation levels of m6A, consequently producing abnormal RNA expression and causing disease. M6A modification is involved in almost all biological processes from normal development to disease, including regulation of the immune system [99]. The normal development and functional exercise of the immune system depend on the precise regulation of related gene expression. Additionally, m6A modification is critical for innate immune responses and anti-tumor immunity [100]. M6A modification is required for a variety of biological activities, including immunological responses, and there is growing evidence that m6A dysfunction is linked to autoimmune disorders.

More studies are needed to determine the mechanism through which m6A methylation genes act in the occurrence and progression of autoimmune diseases. Previous research demonstrated that modifying lncRNA with m6A can change its spatial structure and stability, hence regulating m6A’s RNA and protein binding capabilities [101]. M6A inhibits innate immunity by modifying circRNA. Moreover, the m6A reader YTHDF2 can isolate m6A-circRNA, thereby preventing immune gene activation and adjuvant activity [102]. However, there are few studies on the interactions between ncRNA and m6A in autoimmune diseases. In addition, T cell homeostasis and differentiation are impaired in the absence of METTL3 in mouse T cells. M6A is associated with the control of T cell homeostasis and IL-7-induced differentiation [103]. New pathways for m6A in T cell-mediated pathogenesis, T cell homeostasis, and the differentiation of other immune cells need to be further explored. The intervention of drugs in m6A methylation remains insufficient. Therefore, further studies are needed to identify potential targets and molecular mechanisms of m6A in autoimmune diseases.

Although m6A methylation has been the focus of research in recent years, our understanding of this process is far from complete. The specificity between the m6A sequence and the position and level of reading-protein methylation is unclear. The competition and cooperation between different m6A methylation-modifying enzymes need to be further elucidated. Furthermore, in the course of treatment, traditional Chinese medicine compounds have multi-component, multi-target, and multi-pathway characteristics. Ultimately, m6A has its own characteristics and advantages in the prevention and treatment of complex autoimmune diseases. These advantages offer the possibility for herbal compounds to interact with m6A methylation-modifying enzymes. However, whether key compounds in plants affect disease progression by binding or inhibiting methylation-modifying enzymes remains to be determined.

The specific biological function of m6A methylation may make it an ideal biomarker for clinical applications. Although numerous studies have demonstrated abnormal levels of certain proteins associated with m6A methylation in RA and SLE patients, the abnormal expression of these proteins needs to be further verified. There is also evidence to support the significance of m6A modification in autoimmune diseases such as RA and SLE, for which m6A modification represents a potential molecular target for diagnostics and treatment. However, whether the modification of m6A and related genes or enzymes can be used as drug targets or diagnostic indicators remains to be confirmed.

## Data Availability

Not applicable.

## Abbreviations

AITD: Autoimmune diseases mainly include hyperthyroidism
anti-dsDNA: Anti-double-stranded DNA
anti-ENAs: Anti-extracted nuclear antigen
BLM: Bleomycin
FLS: Fibroblast-like synoviocytes
LncRNA: Long noncoding RNA
Mrna: Messenger RNA
miRNA: MicroRNA
MS: Multiple sclerosis
SMOC2: Modular calcium binding protein 2
MYO1C: Motor protein myosin 1c
m6A: N6-methyladenosine
ncRNA: Non-coding RNA
PBMC: Peripheral blood monocytes
PTEN: Phosphatase and tensin homolog deleted on chromosome 10
pSS: Primary Sjogren's Syndrome
PMS: Progressive multiple sclerosis
RRMS: Relapsing–remitting multiple sclerosis
RA: Rheumatoid arthritis
SNPs: Single nucleotide polymorphisms
SLE: Systemic lupus erythematosus
Sar: Sarsasapogenin
STZ: Streptozotocin
T1DM: Type 1 diabetes
TP: Triptolide
